# Identification of Chiari Type I Malformation subtypes using whole genome expression profiles and cranial base morphometrics

**DOI:** 10.1186/1755-8794-7-39

**Published:** 2014-06-25

**Authors:** Christina A Markunas, Eric Lock, Karen Soldano, Heidi Cope, Chien-Kuang C Ding, David S Enterline, Gerald Grant, Herbert Fuchs, Allison E Ashley-Koch, Simon G Gregory

**Affiliations:** 1 Duke Center for Human Genetics, Duke University Medical Center, Durham, NC, USA; 2 Duke Center for Human Disease Modeling, Duke University Medical Center, Durham, NC, USA; 3 Division of Neuroradiology, Department of Radiology, Duke University Medical Center, Durham, NC, USA; 4 Department of Neurosurgery, Stanford University/Lucile Packard Children’s Hospital, Stanford, CA, USA; 5 Division of Neurosurgery, Department of Surgery, Duke University Medical Center, Durham, NC, USA; 6 Duke Molecular Physiology Institute, Duke University Medical Center, Durham, NC, USA

**Keywords:** Chiari Type I Malformation, Posterior fossa, Disease subtypes, Whole genome expression, Cranial base morphometrics, Clustering

## Abstract

**Background:**

Chiari Type I Malformation (CMI) is characterized by herniation of the cerebellar tonsils through the foramen magnum at the base of the skull, resulting in significant neurologic morbidity. As CMI patients display a high degree of clinical variability and multiple mechanisms have been proposed for tonsillar herniation, it is hypothesized that this heterogeneous disorder is due to multiple genetic and environmental factors. The purpose of the present study was to gain a better understanding of what factors contribute to this heterogeneity by using an unsupervised statistical approach to define disease subtypes within a case-only pediatric population.

**Methods:**

A collection of forty-four pediatric CMI patients were ascertained to identify disease subtypes using whole genome expression profiles generated from patient blood and dura mater tissue samples, and radiological data consisting of posterior fossa (PF) morphometrics. Sparse k-means clustering and an extension to accommodate multiple data sources were used to cluster patients into more homogeneous groups using biological and radiological data both individually and collectively.

**Results:**

All clustering analyses resulted in the significant identification of patient classes, with the pure biological classes derived from patient blood and dura mater samples demonstrating the strongest evidence. Those patient classes were further characterized by identifying enriched biological pathways, as well as correlated cranial base morphological and clinical traits.

**Conclusions:**

Our results implicate several strong biological candidates warranting further investigation from the dura expression analysis and also identified a blood gene expression profile corresponding to a global down-regulation in protein synthesis.

## Background

Chiari Type I Malformation (CMI), characterized by the downward displacement of the cerebellar tonsils through the foramen magnum at the base of the skull, has an estimated prevalence in the United States of slightly less than one percent [1,2]. While there is currently a lack of consensus regarding diagnostic criteria, patients are usually diagnosed if both tonsils are herniated 3 mm or more or one tonsil is herniated 5 mm or more. Patients can either experience a wide range of debilitating neurologic symptoms, with the hallmark symptom consisting of occipital headaches triggered by Valsalva maneuver, or be asymptomatic. Currently, the only treatment for CMI without hydrocephalus is posterior fossa (PF) decompression surgery that expands the posterior fossa, allowing more room for the cerebellar tonsils and achieving improved cerebrospinal fluid flow.

Although patients may be diagnosed with CMI without additional classification, disease presentation is highly variable among patients with respect to symptom presentation, including presence of associated conditions, and the extent of cerebellar tonsillar herniation. Age of onset is also varied with some patients diagnosed shortly after birth while others are diagnosed well into adulthood. Even response to surgery is variable, with some patients achieving significant relief from headaches and associated neurologic symptoms, while others continue to exhibit symptomology post-surgery. In addition to clinical heterogeneity, multiple distinct biological mechanisms have been proposed for tonsillar herniation including cranial constriction, cranial settling, spinal cord tethering, intracranial hypertension, and intraspinal hypotension [3]. The “cranial constriction” mechanism, which is generally thought to represent “classical” CMI, is believed to result from underdeveloped occipital bones resulting in a posterior fossa (PF) that is too small to accommodate the normal sized cerebellum [4,5]. This clinical, mechanistic, and likely etiologic variability coupled with the fact that no consistent diagnostic criteria exist for CMI, pose significant challenges in identifying the genetic basis of the disease and call for approaches that dissect and account for this heterogeneity.

Despite the fact that several lines of evidence exist supporting a genetic contribution to CMI such as twin studies, familial clustering, and co-segregation with known genetics syndromes (Reviewed in [6]), limited research has been conducted to identify the specific genetic factors involved. The first whole genome screen, conducted in 2006, reported significant evidence for linkage to regions on chromosomes 9 and 15 using 23 non-syndromic, CMI multiplex families [7]. There has been one published case–control candidate gene association study that identified four SNPs in CDX1, FLT1, ALDH1A2 that were significantly associated (FDR < 0.10) with CMI when the study population was restricted to those 186 patients determined to have a small posterior fossa [8]. Our group has subsequently carried out two additional whole genome screens. In the first screen, we used 66 non-syndromic, CMI multiplex families and conducted a stratified linkage analysis using clinical criteria to reduce heterogeneity [6]. This approach resulted in a marked increase in evidence for linkage to multiple regions of the genome, including chromosomes 8 and 12, both of which contain growth differentiation factors (GDF6 and GDF3, respectively). Growth differentiation factors have been previously implicated in Klippel-Feil syndrome [9-11], presenting in about 3-5% of CMI patients [12,13]. In the second genome screen, we conducted an ordered subset analysis (OSA) using heritable, disease-relevant PF traits to identify increased evidence for linkage within subsets of families that were similar with respect to cranial base morphological traits [14]. Results from OSA identified multiple genomic regions showing increased evidence for linkage, including regions on chromosomes 22 and 1 which implicated several strong biological candidates for disease [14].

While the motivation for the present study is similar to our two recent CMI genetic studies, the approach adopted here differs substantially. In the previous studies, patients were stratified into homogeneous groups based only on observable phenotypic differences (e.g. clinical criteria or posterior fossa traits) with the goal of reducing genetic heterogeneity and increasing power to identify disease genes within etiologically more similar strata. In the present study, an unsupervised approach was used to define subtypes within a case-only population of unrelated individuals. Furthermore, in addition to the use of cranial base morphological traits, whole genome expression profiles from CMI patient tissue and blood samples were used to cluster patients to establish disease subtypes.

Appropriate tissue selection for gene expression analysis can be difficult, especially for what is considered to be a developmental disorder in most cases and one in which limited knowledge exists about the underlying biological mechanism. While both the age and source of the tissue can influence gene expression, there are a limited number of non-postmortem tissues that can be examined for CMI due to accessibility. One of the most easily obtainable tissues (dura mater) during a standard CMI decompression surgery with duraplasty was therefore selected for whole genome expression analysis. The dura mater is the outermost meningeal layer surrounding the brain and spinal cord. In addition to ease of collection from surgical patients, dura mater may also be relevant to CMI as previous studies have reported the presence of a thickened dural band at the cranio-vertebral junction of CMI patients [15,16], which shows evidence of increased collagen fiber splitting and branching, as well as hyalinosis, calcification, and ossification [15]. Furthermore, examination of the transcription profile of this tissue can shed additional light regarding its potential relevance since the expression profile of dura has not been previously examined in this context. Finally, the comparison of blood vs. dura expression profiles may provide insight regarding the usage of potential biomarkers in blood for the identification of patient subsets.

The present study uniquely allows us to examine disease heterogeneity using biological and radiological data both individually, as well as collectively, to define homogeneous classes of patients or subtypes. The use of biological data alone allows us to identify pure biological subtypes, yet still be able to correlate them with clinical and radiological traits for additional interpretation and characterization. In comparison, the integration of biological and radiological data during the clustering analysis places a greater emphasis on genes and PF traits that are related and collectively establish disease subtypes. The ultimate goal of this study is to gain insight into what factors may be driving disease heterogeneity and to aid in the identification of potential genetic factors that contribute to the development of CMI.

## Methods

### Study population

Study participants were less than 18 years of age, diagnosed with CMI, and had a malformation severe enough to warrant PF decompression surgery with duraplasty. Eligible study participants were identified over a period of 1 year and 8 months through the pediatric neurosurgery practices at the Duke University Medical Center (H.F., G.G.). Participation in the study involved signing consent forms for the release of medical records and pre-surgical brain MRIs, providing a blood and dura sample for RNA extraction, and completing a clinical questionnaire. A total of forty-four pediatric CMI patients were included in this study and detailed study population characteristics are provided in Table 1. The participation rate was high, with only 7.9% of eligible participants declining enrollment. Parents of all minor children and children aged 12 and above provided written informed consent for participation in the study that had been approved by the institutional review board of Duke University Medical Center.

**Table 1:** Study population description

**Description**	**N**	**Percentage**
**Total number of individuals**	44	
**Sex**		
Male	28	63.6%
Female	16	36.4%
**Race**		
White	31	70.5%
African American	13	29.6%
**Syrinx**		
Yes	10	22.7%
No	34	77.3%
**Family history**		
Yes	6	13.6%
No	36	81.8%
Unknown	2	4.6%
**Datasets**		
Blood gene expression	44	100.0%
Dura gene expression	44	100.0%
Cranial	40	90.9%
Clinical questionnaire	36	81.8%
**Age at surgery (years)**^**a**^	8.89 ± 5.19	

^a^Average age at surgery ± standard deviation.

### Posterior cranial fossa measurements

**Figure 1:**
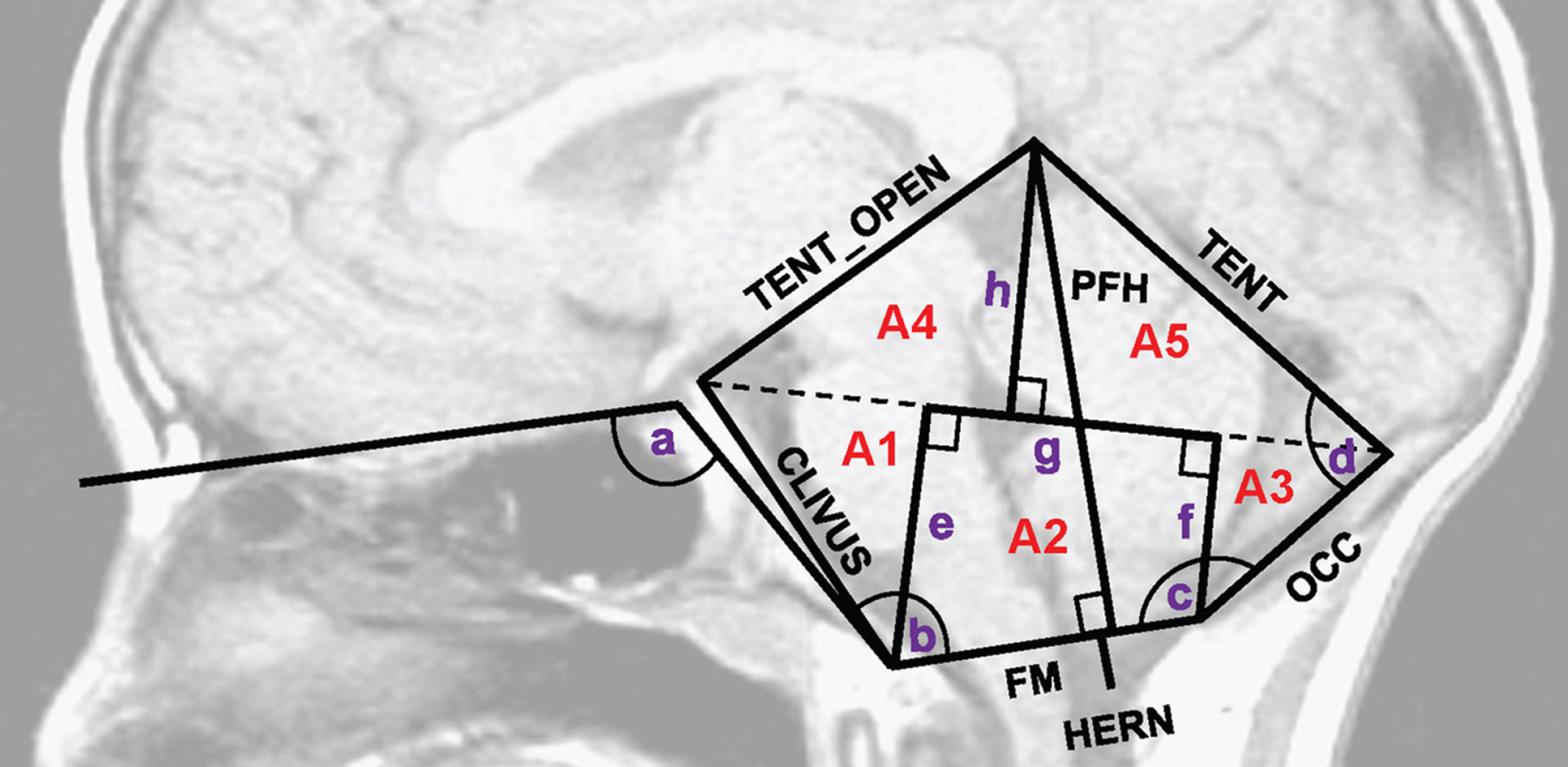
**Posterior cranial fossa measurements taken from the midline of a sagittal, T1-weighted MRI.** A1 to A5 shown in red indicate the regions in which area was estimated. Additional measurements not explicitly labeled include **(a)** basal angle (BASAL_ANG), **(b)** boogaard angle (BOOG_ANG), **(c)** occipital angle (OCC_ANG), **(d)** tentorial angle (TENT_ANG), **(e)** basion to reference (BASTOREF), **(f)** opisthion to reference (OPISTOREF), **(g)** trapezoid height (TRAPHEIGHT), and **(h)** tentorium to reference (TENTTOREF). Abbreviations: TENT_OPEN = tentorial opening, TENT = tentorium, OCC = supraoccipital bone, FM = foramen magnum, PFH = posterior fossa height, and HERN = cerebellar tonsillar herniation. This figure is reproduced from [14], a Wiley publication.

Forty of the forty-four individuals had pre-surgical T1-weighted sagittal brain MRIs available for measurements of the PF region. In total, 18 PF measurements were taken and 8 separate PF areas were estimated per individual (Figure 1) as has been previously described in detail [14]. All measurements were performed by one trained researcher and then confirmed by a board certified neuroradiologist (D.E.). Measurements were taken for right and left herniation (also computed minimum and maximum herniation), as well as the foramen magnum, tentorium, supraoccipital bone, clivus, tentorial opening, PF height, basion to reference line, opisthion to reference line, tentorium to reference line, trapezoid height, tentorial angle, occipital angle, basal angle, Boogaard’s angle, PF area, PF area above the reference line, PF area below the reference line, and areas 1–5 as described previously (Figure 1) [14].

### Clinical questionnaire

A clinical questionnaire collecting information regarding pre-surgical symptom presentation, pregnancy history, presence of associated conditions, and family medical history was provided to participants. The questionnaire was sent to participants post-surgery and was completed either in a web-based or paper-based format, or administered over the phone by study personnel. Thirty-six of the forty-four individuals completed the questionnaire at least partially and, in most cases, the parent was the informant.

### Laboratory protocols

#### Sample collection and storage

All biological samples were collected during PF decompression surgery performed by one of two pediatric neurosurgeons (G.G. or H.F). The standard decompression surgery involves a craniectomy followed by the creation of a “Y” shaped dural opening spanning from the suboccipital region down to the bottom of the cerebellar tonsils. The dura is then closed with a cadaveric pericardial patch to expand the subarachnoid space beneath. During this procedure (duraplasty), a small piece of dura mater (<5 mm × 5 mm) was obtained from either the superior or lateral flap in the cranial portion of the dural opening and immediately stored in a tube filled with 1.25 ml of RNALater (Life technologies, Grand Island, NY) at room temperature. The tube was then placed at 4°C for 24 hours before it was moved to -20°C for long term storage. In addition to the collection of dura, blood was collected in a 2.5 ml Paxgene RNA tube (Qiagen, Valencia, CA) under anesthesia from an arterial line for intraoperative monitoring and blood draws. The Paxgene RNA tubes were incubated at room temperature for 2 hours and then transferred to -20°C for long term storage.

#### RNA extractions

Although patient ascertainment lasted for over a year, all samples were extracted within one month following the completion of ascertainment. RNA was extracted from the dura using the Qiagen fibrous tissue mini kit (Valencia, CA) per the manufacturer’s protocol. Using the OmniBead Ruptor 24 - Bead Mill Homogenizer (Omni International, Kennesaw, GA), the dura samples were first homogenized at 4°C in 2 ml Omni bead ruptor tubes prefilled with 2.38 mm metal beads and buffer RLT (Qiagen, Valencia, CA) plus β-Mercaptoethanol. The following machine settings were used to homogenize all samples: Speed of 6.3 m/s, 2 cycles of 30 seconds, and a 30 second dwell. After extraction, a DNAse digestion of the RNA eluate was performed followed by clean-up using the Qiagen Fibrous Tissue Mini kit (Valencia, CA) according to the manufacturer’s protocol for the Qiagen RNeasy Mini kit (Valencia, CA). RNA was extracted from the blood using the PAXgene Blood RNA kit (Qiagen, Valencia, CA) per the manufacturer’s protocol. During the protocol an on-column DNAse digestion was performed. Purified RNA samples from both the blood and dura were stored in multiple aliquots at -80°C in order to minimize the number of freeze-thaw cycles.

The Nanodrop (ThermoScientific, Wilmington, DE) was used to quantify the RNA and the Agilent RNA 6000 Pico chip (Santa Clara, CA) was used to determine a final concentration and assess quality using the RNA Integrity Number (RIN). RNA samples were required to have a RIN exceeding 6 and a total yield of at least 50 ng for further processing.

#### Whole genome expression arrays

Prior to running Illumina HT-12 v4 Expression BeadChips (San Diego, CA), high quality RNA was amplified and converted to biotin-labeled cRNA. All RNA samples were first concentrated using a vacuum centrifuge at 35°C in order to obtain the necessary starting concentration for the Illumina TotalPrep-96 RNA Amplification Kit (San Diego, CA). The protocol was followed according to the manufacturer’s instructions and, in addition to the processing of patient RNA samples, three additional controls were included in the 96 well plate: a positive control included in the Illumina TotalPrep kit (San Diego, CA), a dura control sample (Clontech human dura matter total RNA), and a blood control sample (Clontech human blood, peripheral leukocytes total RNA). Quantification of the cRNA was performed using the Nanodrop (ThermoScientific, Wilmington, DE). An RNA 6000 Pico chip (Agilent, Santa Clara, CA) was run using a subset of representative cRNA samples with varying yield in order to assess the overall size distribution. cRNA samples were then diluted to a concentration of 150 ng/ul and run on Illumina HT-12 v4 Expression BeadChips (San Diego, CA) according to the manufacturer’s protocol. In total, eight chips were run in one experimental batch that included forty-four blood RNA patient samples, forty-four dura RNA patient samples, and one blood and one dura control RNA sample both run in quadruplicate. An attempt was made to distribute samples evenly across the chips according to race, age at surgery, sex, and surgeon. Dura samples were run across four chips with the same dura control sample run on each chip. Similarly, blood samples were run across the remaining four chips with the same blood control sample run on each chip. Sample groups by chip and specific chip positions were kept consistent across the blood and dura chips.

#### Real-time quantitative PCR

Differential expression of six genes was validated using real-time quantitative PCR (RT-qPCR). Following the manufacturer’s protocol, the SuperScript III First-Strand Synthesis System for RT-PCR (Invitrogen, Grand Island, NY) was used to synthesize first strand cDNA from high quality RNA. PCRs were performed using the Qiagen HotStarTaq Plus kit (Valencia, CA) in order to confirm the conversion of RNA to cDNA (β-actin primers provided in the Invitrogen kit) and that no genomic DNA was present (custom primers were used that targeted a non-coding region of the genome). In order to identify appropriate endogenous controls to use for RT-qPCR, Human Taqman Endogenous Control Arrays (Applied Biosystems, Grand Island, NY) were run using blood and dura cDNA samples. SASqPCR [17] was used to identify the most stable endogenous control genes. Due to sample limitations, only the top two most stable genes (lowest M value) were selected from the blood and dura RT-qPCR results. Taqman gene expression assays with TaqMan Gene Expression Master Mix (Applied Biosystems, Grand Island, NY) were used to assess the gene expression of three target genes in dura (NOTCH4: Hs00965895_g1, ETS1: Hs00428293_m1, ETS2: Hs01036305_m1), three target genes in blood (PRPF38B: Hs00216242_m1, PSMA3: Hs00160558_m1, RSL24D1: Hs00829770_g1), two endogenous control genes in dura (PPIA: Hs99999904_m1, ACTB: Hs99999903_m1), and two endogenous control genes in blood (HMBS: Hs00609297_m1, PGK1: Hs99999906_m1). Due to RNA availability, a limited number of samples were assessed. After matching for important covariates (see Data analysis below), ten cDNA samples from each blood class (N_total_ = 20) and five cDNA samples from each dura class (N_total_ = 10) were selected for the validation experiments. All reactions were performed in triplicate (0.5 μl of 20X TaqMan Gene Expression Assay, 5 μl of 2X TaqMan Gene Expression Master Mix, 2 ng blood cDNA or 1.5 ng dura cDNA, water to bring the reaction volume to 10 ul) and run on a ViiA™ 7 Real-Time PCR System (Applied Biosystems, Grand Island, NY); blood and dura samples were run on separate 384 well plates.

### Data analysis

#### Whole genome expression quality control and data pre-processing

Initial quality assessment of the whole genome expression data was performed using Illumina’s GenomeStudio Gene Expression module (San Diego, CA) for the blood and dura samples separately. Illumina system controls were checked for consistency with expected performance. Additional control metrics such as the number of detected genes (based on Illumina’s detection p-value), signal intensity measures, housekeeping gene intensity, and several sample-independent system control metrics were assessed for each sample to identify outliers as defined by greater than 4 standard deviations away from the mean. In addition, technical control replicates were assessed for consistency.

Raw expression data were log2-transformed followed by quantile normalization using the R package, lumi [18]. The least variable probes as defined by the 75^th^ percentile of the distribution of coefficient of variation (CV) values were removed to reduce noise using R 2.15.0 (N_ProbesRemaining_ = 11804). In order to identify sample outliers and assess sample relationships, principal components analysis (PCA) was performed using prcomp in R 2.15.0 and samples were plotted using the first two PCs. This was performed both with and without the inclusion of control blood and dura technical replicates. Sex of the samples was confirmed by assessing the Y chromosome gene expression probes and confirming that samples clustered on the basis of reported sex after running PCA using prcomp in R 2.15.0.

#### Sparse k-means clustering

Prior to clustering analyses, studentized residuals were calculated for each expression probe from linear regression models adjusting for age at surgery, sex, neurosurgeon, and race using SAS 9.3 (Cary, NC) so that sample classes would not be identified solely based on these factors. Completion of a preliminary clustering analysis led to the observation that initial dura RNA quality assessed by the RIN was associated with some of the identified classes (data not shown). Thus, the dura gene expression data were further adjusted by also including the RIN as a covariate in the linear regression models. In order to exclude the effects of age, sex, and race on MRI measurements, each PF trait was regressed on age at MRI, race, and sex and only the studentized residuals were considered for the clustering analysis. In order to avoid missing values, left and right herniation were removed from the analysis (N_RemainingPFtraits_ = 24).

Sparse k-means clustering [19] was used to identify patient subtypes or classes from the blood whole genome expression data, dura whole genome expression data, and PF trait data separately. Standard k-means clustering groups objects into a pre-defined number of k classes without feature selection such that the within sum of squares is minimized. Sparse k-means clustering differs from the standard approach in that it adaptively selects a subset of features (e.g. gene expression probes or PF traits) to cluster the objects (e.g. patients). Specifically, a weighted between cluster sum of squares (BCSS) is maximized conditional on feature weight restrictions. Note that when all features are given equal weights sparse k-means clustering reduces to standard k-means clustering. In sparse k-means clustering each feature is given a non-negative weight and depending on the magnitude of the tuning parameter, a proportion of the features will be given a weight of zero indicating that they do not contribute to the clustering (i.e. sparsity was enforced). The optimal tuning parameter and number of k classes are determined by identifying which combination produces the largest gap statistic which assesses the overall strength of the clustering compared to clustering of the data when the objects are independently permuted within each feature (null data) [19]. More specifically, the gap statistic represents the logarithm of the difference between the observed BCSS and the expected BCSS. In general, sparse clustering has a number of potential advantages including increased interpretability of findings as a result of feature specific weights and a reduction in noise leading to tighter clusters due to the removal of features that do not contribute to class discrimination.

Prior to the clustering analysis, arrays were first standardized (μ = 0, σ = 1). The R package, sparcl [20], was then used to implement sparse k-means clustering using the squared Euclidean distance as the dissimilarity measure between objects, 20 random starts and a maximum of 20 iterations of the k-means algorithm. For each value of k tested (k = 2-5), 50 tuning parameter values were assessed. The optimal k/tuning parameter combination was determined based on the maximal gap statistic (N_permutations_ = 25). Increasing the number of permutations to 100 did not substantively alter the results. Assuming the gap statistic follows a standard normal distribution, an approximate p-value (pnorm function in R 2.15.0) and a 95% confidence interval (CI) were generated using R 2.15.0. In order to visualize the sample classes determined from the sparse k-means clustering analysis, PCA using prcomp in R 2.15.0 was performed using weighted features as input; feature weights were determined from the sparse k-means analysis.

#### Integrative sparse k-means clustering

The identification of patient subtypes or classes were also determined from the following analyses integrating multiple data types: 1) Dura gene expression and PF trait data, 2) Blood gene expression and PF trait data, and 3) Dura gene expression, blood gene expression, and PF trait data. Sparse k-means clustering was performed as described above, but with modifications to accommodate the heterogeneity of these datasets. The modifications for the integrated analysis were as follows: 1) Before clustering, each dataset was scaled to have the same total weighted sum of squares (within cluster sum of squares plus between cluster sum of squares). This ensures that the clustering would not simply be driven by the larger dataset, 2) The tuning parameter that controls the level of sparsity was adjusted relative to the number of features in each dataset, and 3) The gap statistic was used as described above, except that objects were permuted within each dataset rather than within each feature. The purpose of this approach was to identify clusters that are significantly expressed across multiple datasets, rather than multiple features expressed in a single dataset. In other words, integrative clustering relies on joint structure across datasets, rather than individual dataset structure. To be consistent with the sparse k-means clustering applied to individual datasets, k values of 2 through 5 each with 50 tuning parameter values were assessed for each of the integrated analyses. Inference for the gap statistic, as well as the visualization of sample relationships and class membership using weighted PCA was performed as described above.

The R functions used to implement integrative sparse k-means clustering are available upon request. In addition, a more mathematically detailed description of the integrative approach is provided (see Additional file 1).

#### Class characterization

Concordance across analyses with respect to class membership was assessed using a Rand index (range is 0 to 1) and an adjusted Rand index (range is -1 to 1) as implemented in the R package, fossil [21], using R 2.15.0. These both provide a measure of agreement between two clustering outcomes or data partitions, while the adjusted Rand index provides more sensitivity with its increased range and also accounts for randomness in class assignment [22]. Analyses were restricted to those forty individuals present in all datasets used for clustering.

Clinical characterization of classes was determined using the clinical questionnaire provided to participants as well as medical records in a few cases (presence of hydrocephalus and syrinx). With the exception of a few continuous variables in the questionnaire, all analyses were performed using a Fisher’s exact test as implemented in SAS 9.3 (Cary, NC) to determine which clinical features were associated with class membership. Continuous variables were tested for association with class by using a *t*-test assuming equal or unequal variance, when appropriate, or ANOVA when the number of classes exceeded 2 (SAS 9.3, Cary, NC). Prior to analysis, all continuous variables were tested for normality using the SAS procedure, proc univariate (Shapiro-Wilk and Kolmogorov-Smirnov test). If necessary, variables were transformed to approximate a normal distribution.

Biological characterization of classes was based primarily on the gene expression probe weights assigned from the sparse k-means or integrative sparse k-means clustering analysis. As no gene expression data were used to identify classes solely dependent on PF traits, another approach was necessary to biologically characterize these classes. The R package, limma [23], was used to identify gene expression probes in blood and dura that were differentially expressed between the cranial (PF trait) classes. Log_2_-transformed, quantile normalized expression data were used as input and age at surgery, sex, race, and surgeon were included as covariates in the model. For the dura whole genome expression analysis, the RIN was also included as a covariate in the model for reasons discussed above. Illumina HumanHT12 v4 probe annotation data were pulled from the R annotation package, illuminaHumanv4.db [24]. The top 100 ranked genes from each of the 6 analyses based on either p-values from limma (R package used to identify differentially expressed gene expression probes) or feature weights from sparse k-means or integrative sparse k-means clustering were used as input into DAVID v6.7 [25,26] to identify enrichment for KEGG biological pathways. The re-annotation of Illumina probes was used to create a custom background of genes for the pathway enrichment analysis. In order to assess enrichment for specific biological pathways, DAVID v6.7 [25,26] implements both a Fisher’s exact test and a modified Fisher’s exact test (EASE score) which is more conservative as the number of genes provided in the user list that are also found in the pathway of interest is reduced by one. In addition, Benjamini-Hochberg corrected p-values are provided in order to account for multiple testing.

Radiological characterization of classes was mostly based on PF feature weights provided by sparse k-means or integrative sparse k-means clustering. For the classes determined based solely on dura gene expression or blood gene expression data, a separate approach was necessary. Association analyses were therefore carried out using logistic regression in SAS 9.3 (Cary, NC) with age at MRI, sex, and race included as covariates in the models. For each of the clustering analyses, the PF traits most associated with the identified classes will be reported.

#### Real-time quantitative PCR

SAS 9.3 (Cary, NC) was used to perform the RT-qPCR analysis. Prior to analysis, sample outliers as defined by greater than 4 standard deviations away from the mean were first identified by gene (comparison to other samples and comparison across technical replicates within a sample). Standard RT-qPCR analyses were conducted [27]: 1) For each sample-gene combination, the arithmetic mean C_T_ of the three technical replicates was calculated, 2) For each sample, the geometric mean C_T_[28] of the two endogenous control gene means was calculated, 3) For each sample-target gene combination, the ΔC_T_ was calculated by subtracting the mean C_T_ of the two endogenous control genes from the mean C_T_ of the target gene, and 4) For each target gene, the ΔC_T_ of class 1 was compared to class 2 using a Wilcoxon rank-sum exact test (one-sided p-value).

## Results

### Whole genome expression sample quality assessment

Quality assessment of blood and dura whole genome expression data was performed separately using multiple approaches as described under the Methods section. For all data, Illumina system controls were first checked and found to be consistent with expected performance. In addition, several control metrics were assessed on an individual sample basis to identify samples with poor overall quality. None of the blood RNA samples were identified as outliers. One of the dura RNA samples was considered an outlier for only 2 out of the 15 control metrics examined (signal average and housekeeping gene intensity) and was thus retained for analysis. In addition, technical control replicates for both blood and dura were assessed for concordance. As expected, the Pearson correlation coefficient was > 0.99 and > 0.98 for the dura and blood control replicates, respectively. Furthermore, when sample relationships were examined using either hierarchical clustering as implemented in lumi [18] (R package used to implement methods for Illumina gene expression data) or PCA, all control replicates clustered with one another and away from the remaining patient samples indicative of their high degree of concordance. In order to further assess sample relationships and detect possible outliers due to technical reasons, PCA was performed using the patient samples without the inclusion of technical control replicates. No samples were removed from the analysis for the following reasons: 1) The samples that clustered away from the others were not considered severe outliers as defined by greater than 4 standard deviations away from the mean PC score, and 2) None of the samples that clustered away from the larger group had obvious technical or quality issues when further examined and may in fact represent something biologically interesting. Final quality assessment consisted of sample sex checks performed as described above in the Methods section. All samples were found to group with other samples of the same reported sex.

### Sparse k-means clustering

**Figure 2:**
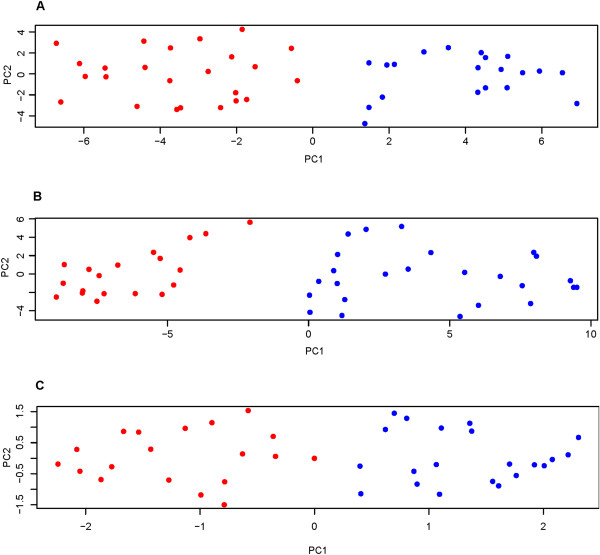
**Weighted PCA plots for individual sparse k-means clustering.** Each point represents a patient and each class of patients is shown in a different color (blue or red). **(A)** Dura (Proportion of variance explained by PC1 (0.26) and PC2 (0.07)), **(B)** Blood (Proportion of variance explained by PC1 (0.46) and PC2 (0.09)), and **(C)** Cranial sparse k-means clustering analysis (Proportion of variance explained by PC1 (0.54) and PC2 (0.18)). The “true” patient class is unknown and is based on the sparse k-means clustering assignment.

Sparse k-means clustering was performed using blood and dura whole genome expression data separately, as well as PF trait data. A summary of the results are shown in Table 2 and sample relationships can be visualized according to class membership in the weighted PCA plots presented in Figure 2. The optimal number of k classes as determined by the gap statistic was two for all three analyses and generated fairly similar class sizes. For the blood and dura whole genome expression clustering analysis, no sparsity was enforced as indicated by the fact that all 11804 gene expression probes received non-zero feature weights. Increased pre-filtering (restriction to the most variable 5000 gene expression probes) did not enforce sparsity either. Sparsity was enforced, however, for the PF trait clustering analysis with 25% of the PF traits assigned weights of zero. Under the assumption that the gap statistic follows a standard normal distribution, approximate p-values for all three analyses were nominally significant (p < 0.05), with the blood and dura analysis resulting in the most significant gap statistics (p < 1×10^-10^). This can also be visualized by the extent of separation observed between the classes based on PC1 from the weighted PCA (Figure 2).

**Table 2:** **Sparse k-means clustering results**^**a**^

**Description**	**Individual clustering**		
	**Dura**	**Blood**	**Cranial**
**Optimal k classes**^**b**^	2	2	2
Class 1 (N)	24	25	21
Class 2 (N)	20	19	19
**Optimal tuning parameter**	68.27	81.71	3.66
**N non-zero weighted features (%)**^**c**^			
Dura	11804 (100%)	NA	NA
Blood	NA	11804 (100%)	NA
Cranial	NA	NA	18 (75%)
**Maximum weighted feature (weight)**			
Dura	MECOM (0.21)	NA	NA
Blood	NA	RPS7 (0.17)	NA
Cranial	NA	NA	BASTOREF (0.72)
**Gap statistic**^**d**^	**1.152 ± 0.013**	**1.834 ± 0.014**	**0.487 ± 0.136**
95% Confidence interval	1.126-1.178	1.807-1.860	0.222-0.753
P-value	<1.0E-10	<1.0E-10	1.62E-04

*Abbreviations*: N: number, MECOM: MDS1 and EVI1 complex locus, RPS7: ribosomal protein S7, BASTOREF: basion to reference line, NA: not applicable.

^a^Clustering results presented using individual datasets (Dura: dura gene expression data, Blood: blood gene expression data, Cranial: PF trait data).

^b^44 individuals were included for the dura and blood gene expression individual clustering analyses; 40 individuals were included for all other analyses.

^c^11804 gene expression probes and/or 24 posterior fossa traits (features) were used as input.

^d^The gap statistic ± standard error is presented. Gap statistics with an approximate p-value less than 0.05 are shown in bold.

### Integrative sparse k-means clustering

**Figure 3:**
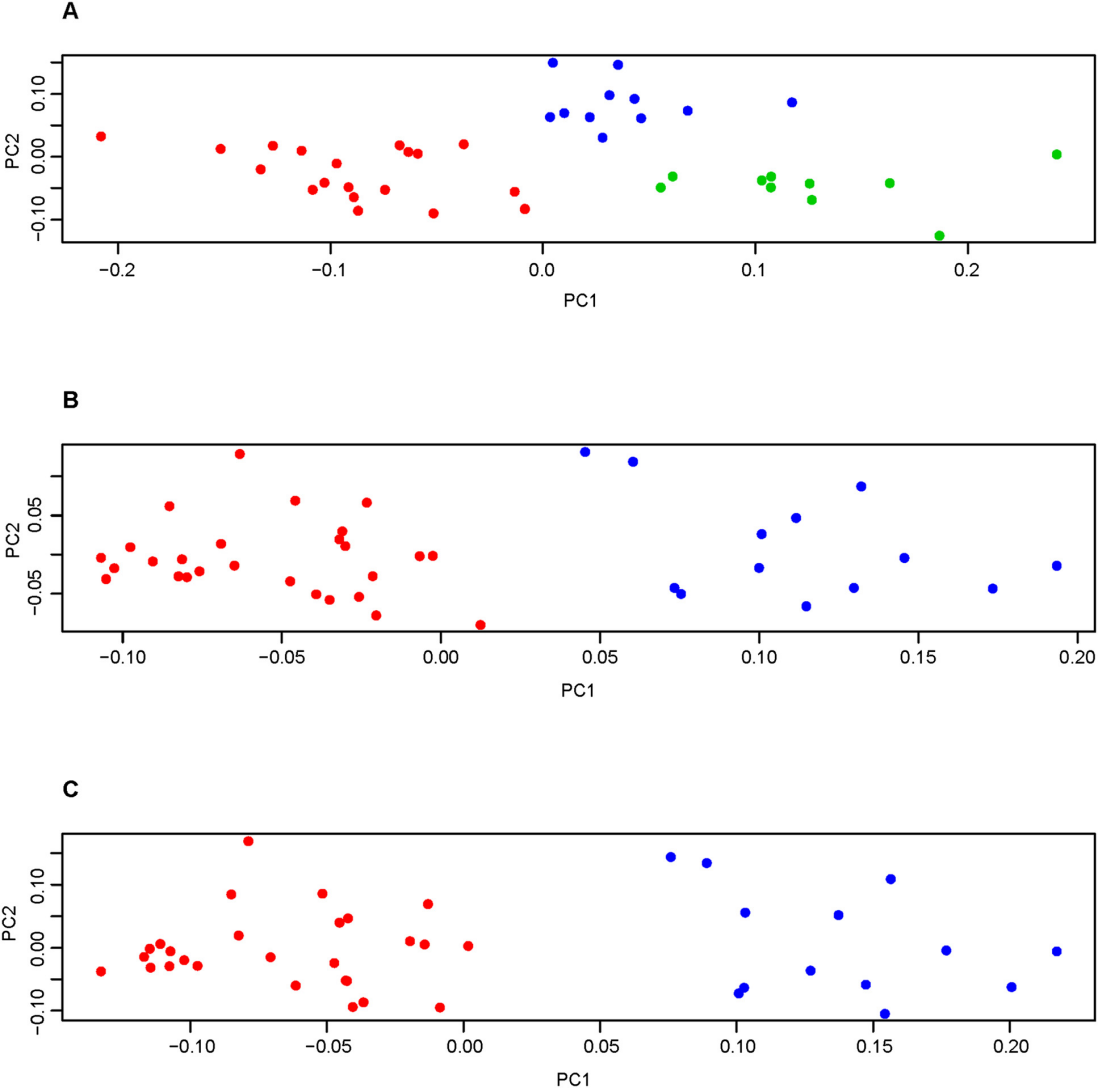
**Weighted PCA plots for integrative sparse k-means clustering.** Each point represents a patient and each class of patients is shown in a different color (blue, red, or green). **(A)** Dura-Cranial (Proportion of variance explained by PC1 (0.39) and PC2 (0.16)), **(B)** Blood-Cranial (Proportion of variance explained by PC1 (0.62) and PC2 (0.24)), and **(C)** Blood-Dura-Cranial integrative sparse k-means clustering analysis (Proportion of variance explained by PC1 (0.40) and PC2 (0.17)). The “true” patient class is unknown and is based on the sparse k-means clustering assignment.

Integrative sparse k-means clustering was performed using 1) Dura gene expression and PF trait data (Dura-Cranial), 2) Blood gene expression and PF trait data (Blood-Cranial), and 3) Blood gene expression, dura gene expression, and PF trait data (Blood-Dura-Cranial). A summary of the results are shown in Table 3 and sample relationships can be visualized according to class membership in the weighted PCA plots presented in Figure 3. The optimal number of k classes as determined by the gap statistic was two for all analyses, except for the Dura-Cranial analysis which defined three classes as optimal. Unlike the results from the individual sparse k-means method, the number of features contributing to class discrimination was restricted for all three integrative analyses (i.e. sparsity was enforced and a proportion of features were assigned weights of zero), with the Blood-Cranial clustering analysis having the fewest number of contributing features. The Blood-Cranial analysis resulted in the most significant gap statistic (p = 2.0×10^-5^) out of the integrative clustering analyses, followed by the Dura-Cranial (p = 7.4×10^-5^) and Blood-Dura-Cranial analysis (p = 3.6×10^-4^).

**Table 3:** **Integrative sparse k-means clustering results**^**a**^

**Description**	**Integrative clustering**		
	**Dura-Cranial**	**Blood-Cranial**	**Blood-Dura-Cranial**
**Optimal k classes**	3	2	2
Class 1 (N)	19	27	27
Class 2 (N)	11	13	13
Class 3 (N)	10	NA	NA
**Optimal tuning parameter**	0.52	0.24	0.35
**N non-zero weighted features (%)**^**b**^			
Dura	6358 (53.9%)	NA	3158 (26.8%)
Blood	NA	1120 (9.5%)	2372 (20.1%)
Cranial	9 (37.5%)	2 (8.3%)	5 (20.8%)
**Maximum weighted feature (weight)**			
Dura	MUC4 (0.24)	NA	LGALS3 (0.34)
Blood	NA	RABGAP1 (0.33)	RABGAP1 (0.26)
Cranial	PFA_TOP (0.73)	BASTOREF (0.99)	BASTOREF (0.89)
**Gap statistic**^**c**^	**0.128 ± 0.034**	**0.302 ± 0.073**	**0.266 ± 0.079**
95% Confidence interval	0.062-0.194	0.158-0.446	0.112-0.420
P-value	7.41E-05	2.01E-05	3.59E-04

*Abbreviations*: *N* number, *MUC4* mucin 4 cell surface associated, *RABGAP1* RAB GTPase activating protein 1, *LGALS3* lectin galactoside-binding soluble 3, *BASTOREF* basion to reference line, *PFA_TOP* posterior fossa area above the reference line, *NA* not applicable.

^a^Dura: dura gene expression data, Blood: blood gene expression data, Cranial: PF trait data.

^b^11804 gene expression probes and/or 24 posterior fossa traits (features) were used as input.

^c^The gap statistic ± standard error is presented. Gap statistics with an approximate p-value less than 0.05 are shown in bold.

### Class characterization

In order to assess class membership concordance, individuals were restricted to the forty present in all analyses and both a Rand and an adjusted Rand index were computed, results of which are summarized in Table 4 (see Additional file 2 for class assignments for all samples for each analysis). Of particular note, the Blood-Cranial and Blood-Dura-Cranial integrative clustering analyses partitioned the patients into the same two classes thus the addition of the dura gene expression data did not alter the class assignments. This would also be consistent with the fact that the Dura with Blood-Cranial and Dura-Blood-Cranial comparisons resulted in the lowest class agreement (Adjusted Rand index = 0.01). With the exception of the Blood-Cranial and Blood-Dura-Cranial comparison, the remaining class membership comparisons showed somewhat limited concordance. The Cranial with Dura-Cranial comparison resulted in the next highest class agreement (Adjusted Rand index = 0.55), followed by Dura with Dura-Cranial (Adjusted Rand index = 0.35).

**Table 4:** **Class membership comparison**^**a**^

**Class 1**	**Class 2**	**Adj rand index**	**Rand index**
Blood-Cranial	Dura-Blood-Cranial	1.00	1.00
Cranial	Dura-Cranial	0.55	0.59
Dura	Dura-Cranial	0.35	0.62
Cranial	Blood-Cranial	0.35	0.67
Cranial	Dura-Blood-Cranial	0.35	0.67
Blood	Dura-Cranial	0.33	0.49
Blood	Blood-Cranial	0.29	0.64
Blood	Dura-Blood-Cranial	0.29	0.64
Blood	Cranial	0.10	0.55
Dura-Cranial	Blood-Cranial	0.04	0.51
Dura-Cranial	Dura-Blood-Cranial	0.04	0.51
Blood	Dura	0.02	0.49
Dura	Cranial	0.02	0.49
Dura	Blood-Cranial	0.01	0.49
Dura	Dura-Blood-Cranial	0.01	0.49

^a^Comparisons were made by restricting the analysis to the 40 individuals present in all datasets.

*Abbreviations*: *Adj* adjusted.

The results generated from the blood and dura whole genome expression individual sparse k-means clustering analyses resulted in the most significant gap statistics (p < 1×10^-10^) and are further described below. However, complete characterization of the remaining classes is provided (see Additional file 3). For each analysis, classes were characterized biologically (KEGG pathway enrichment analysis; the top 100 ranked genes from each analysis were used as input into DAVID v6.7, see Methods section for more details), clinically (clinical questionnaire data and medical records; Fisher’s exact test was used for categorical variables; *t*-test or ANOVA was used for continuous variables), and radiologically (PF traits; for dura and blood individual clustering analyses, logistic regression was used; otherwise, the top ranked weighted traits are listed); nominally significant findings are presented in Table 5 (Blood and dura whole genome expression data), as well as Additional file 3. Due to the extent of missing data for the clinical questionnaire, results from very few clinical traits are presented. Only one of the questions was nominally significant with less than 25 percent missing data (paternal age). This meant that out of the 36 patients that had at least partially completed the questionnaire, one additional individual did not respond to the question concerning paternal age. We recognize that this still represents a large proportion of missing data therefore the results should be interpreted with caution. However, we further investigated the pattern of missing data for this question, and found that the missing data was not significantly associated with blood class (Fisher’s exact test, p = 1).

**Table 5:** **Class characterization summary**^**a**^

**Analysis**	**Characterization**	**Description**	**Class 1**	**Class 2**	**P-val**^**e**^
Dura	Biological^b^	Dorso-ventral axis formation	3/3 Up	3/3 Down	0.001
		Pathways in cancer	4/6 Up	4/6 Down	0.031
	Radiological^c^	Area3	Larger	Smaller	0.006
		Supraoccipital bone	Larger	Smaller	0.006
		Opisthion to reference	Larger	Smaller	0.046
	Clinical	NA	NA	NA	NA
Blood	Biological^b^	Ribosome	10/10 Up	10/10 Down	2.80E-09
		Spliceosome	5/6 Up	5/6 Down	0.005
		Proteosome	3/3 Up	3/3 Down	0.007
		RNA degradation	2/3 Up	2/3 Down	0.014
		Oxidative phosphorylation	2/4 Up	2/4 Down	0.018
	Radiological^c^	Boogaard’s angle	Smaller	Larger	0.004
		Basion to reference	Larger	Smaller	0.016
		Tentorium	Smaller	Larger	0.036
	Clinical^d^	Paternal age	Younger	Older	0.021

^a^Only nominally significant results are shown. In addition, only the two most significant clustering analyses were included in the table.

^b^The top 100 ranked genes from each analysis were used as input into DAVID v6.7 for the pathway analysis. KEGG pathways with a Fisher exact p < 0.05 are listed. Additional filtering was applied using DAVID's default settings: minimum of 2 genes present in the pathway and an EASE score < 0.1. For each class, the total number of genes present in each pathway and whether they are down- or up-regulated with respect to the other class are noted.

^c^Logistic regression was carried out including age at MRI, sex, and race as covariates in the model.

^d^A *t*-test assuming equal variance was performed.

^e^These are not adjusted for multiple testing.

For the dura clustering analysis (Table 5), enrichment for two biological pathways was observed, dorso-ventral axis formation and pathways in cancer; neither met adjustment for multiple testing. Radiological characterization of the dura classes indicated that the lower right hand region of the PF appeared to be most different between the identified classes (supraoccipital bone, opisthion to reference, and area3). All of these PF traits were previously found to be heritable in a collection of CMI families [14]. Interestingly, down-regulation of all three genes present in the dorso-ventral axis formation pathway (ETS1 (v-ets erythroblastosis virus E26 oncogene homolog 1 (avian)), ETS2 (v-ets erythroblastosis virus E26 oncogene homolog 2 (avian)), NOTCH4 (notch 4)) was observed in class 2 where patients also exhibited a smaller supraoccipital bone, opisthion to reference line, and area3.

For the blood clustering analysis (Table 5), which also resulted in the most significant gap statistic, enrichment for five biological pathways was detected including the ribosome, spliceosome, proteasome, RNA degradation, and oxidative phosphorylation pathways. Of particular note, the only pathway that remained significant after a Benjamini-Hochberg adjustment for multiple testing was the ribosome pathway (Adjusted p-val = 2.1×10^-6^). Multiple regions of the PF were associated with blood class, including the Boogaard’s angle, tentorium, and the basion to the reference line. Similar to the dura analysis, all of these PF traits were previously found to be heritable in a collection of CMI families [14]. The only clinical association observed with blood class was paternal age; limitations of this analysis were described in detail above. Interestingly, an increased paternal age and an overall down-regulation of ribosome, spliceosome, and proteasome pathways in class 2 compared to class 1 was observed; a slight down-regulation of the RNA degradation pathway was also detected in class 2.

### Real-time quantitative PCR

For the dura RT-qPCR analysis, all three genes assessed were down-regulated in class 2 versus class 1, consistent with the Illumina HT-12 data (see Additional file 4). Both ETS1 and ETS2 were significantly differentially expressed across classes (p < 0.05), while NOTCH4 did not quite meet statistical significance (p = 0.08). For the blood analysis, PSMA3 and RSL24D1 were both significantly down-regulated (p < 0.05) in class 2 versus class 1, consistent with the Illumina HT-12 data (see Additional file 4). However, we were unable to validate the PRPF38B finding. Although not statistically significant, it was up-regulated in class 2 versus class 1 which is the opposite of what was found using the Illumina HT-12 data.

## Discussion

We ascertained forty-four pediatric CMI patients in order to establish disease subtypes using biological data consisting of patient blood and dura whole genome expression profiles, as well as radiological data comprised of PF traits. Sparse k-means clustering analyses were performed using the biological and radiological data both individually and collectively. The latter analysis required us to extend the original sparse k-means method to accommodate multiple datasets from different sources. Identified subtypes were compared across analyses for class membership agreement. Subtypes were also fully characterized for better interpretability, which included the identification of enriched biological pathways, cranial base discriminating features, and, to a more limited extent, correlated clinical traits. All clustering analyses resulted in the identification of significant patient classes, with the dura and blood individual clustering analyses showing the strongest evidence. Further characterization of these classes led to the identification of several factors that may contribute to disease heterogeneity within a pediatric CMI population.

Prior to focusing on our specific findings, we present a general discussion surrounding the results obtained from both the standard and integrative sparse k-means clustering methods. First, limited class membership concordance was observed across most analyses. This was not particularly surprising as the source of each dataset differed substantially (blood vs. dura vs. PF trait). It does, however, suggest that the use of more readily available patient data, such as PF traits, would not be sufficient to identify subtypes established using biological data alone. In addition, the class membership agreement between the dura and blood clustering analyses was extremely low. While each of these analyses identified biological classes, the features that accounted for a large proportion of variation in the data not only differed between the analyses but led to the partitioning of patients into different classes. Specifically, the biological relationships observed among the patients were strongly dependent on the biological source used. While this could be due to our limited sample size or the particular clustering algorithm implemented, it could also be due to the fact that one or both of these tissues were not able to strongly identify true disease subtypes or that each provided us with very distinct information only relevant to the specific tissue assessed. Continued collection of patient samples and further investigation of results would be necessary to resolve these possibilities.

In addition to the individual dataset clustering, we performed integrative clustering. The purpose of these analyses was to place more weight on features within each dataset that act collectively to discriminate patient classes. For example, the Dura-Cranial analysis should identify genes that are relevant to cranial base morphology and able to, in combination with those PF traits, partition patients into distinct subtypes. While this was an attractive a priori approach, the findings did not provide significant insight into disease heterogeneity. In general, the integrative analyses were less significant than the individual clustering analyses. In addition, two out of the three analyses (Blood-Cranial and Blood-Dura-Cranial) did not produce a strong biological interpretation as evident from the lack of enrichment observed for any biological pathway. We thus focus our discussion on the individual clustering analyses, particularly on our two most significant findings: the dura and blood whole genome expression clustering results.

Sparse k-means clustering using blood whole genome expression data alone resulted in our most significant finding. Using the top 100 ranked genes, we identified at least nominally significant enrichment for five biological pathways, including the ribosome, spliceosome, proteasome, RNA degradation, and oxidative phosphorylation pathways. Interestingly, we observed a down-regulation of the ribosome (involved in protein synthesis), spliceosome (involved in splicing of pre-mRNA), and proteasome (involved in the degradation of proteins) pathways in the group of patients that also had older fathers, a smaller basion to reference line, a larger tentorium, and a flatter cranial base as indicated by a larger Boogaard’s angle (Figure 1). One potential link between paternal age and the ribosome comes from a study which examined DNA methylation in rat liver and germ cells and found that a region of the ribosomal DNA (rDNA) locus was preferentially hypermethylated with increased age in both the sperm and liver [29]. Hypermethylation of the rDNA may compromise function and affect protein synthesis [29]. While this study did not directly examine whether this age-dependent methylation change could be passed on to and remain in offspring, aberrant methylation was suggested as a potential mechanism contributing to paternal age related abnormalities in offspring [29]. In a study examining whole genome expression profiles from peripheral blood lymphocytes in children with ASD and controls, decreased variance in the distribution of gene expression levels in children with ASD as well as control individuals with older fathers was reported [30]. This decreased variance was suggested to be due to a global down-regulation of transcriptional regulation [30]. Thus, paternal age appears to be associated with global changes in transcriptional regulation and protein synthesis, which we may be detecting in our blood classes.

Sparse k-means clustering using dura whole genome expression alone resulted in our second most significant finding. Within class 2 we observed a reduction in area3, the supraoccipital bone, and the opisthion to reference line, as well as down-regulation of all genes identified in the enriched dorso-ventral axis formation pathway and over half of the genes identified in pathways in cancer. While these pathways are very general and don’t appear immediately relevant, several of the genes involved in these pathways have multiple functions, some of which are applicable to CMI biology.

Two of the genes identified within the dorso-ventral axis formation pathway, ETS1 and ETS2, are involved in osteoblast differentiation and the formation of bone [31]. While some studies have suggested that overexpression of ETS2 is associated with the craniofacial abnormalities observed in Down syndrome patients [32], Hill and colleagues found that, in general, trisomy 16 mice (model for human trisomy 21, Down syndrome) with or without an extra copy of ETS2 produced comparable craniofacial abnormalities [33]. However, this study did observe a more severely shortened occipital bone in trisomy 16 mice with only two (16% reduction) versus three copies of ETS2 (4% reduction) when compared to euploid mice. This suggests a more complex interplay among genes present within the Down syndrome interval. Another potential link between ETS1/2 and cranial abnormalities comes from a recent report that found that reduced expression of Ets2 repressor factor (ERF) causes complex craniosynostosis characterized by premature fusion of multiple cranial sutures, craniofacial abnormalities, language delay, and CMI [34]. Although not one of our most significant findings, reduced expression of ERF was also observed in class 2 (adjusted p = 0.04). The third gene identified within the dorso-ventral axis formation pathway is NOTCH4. NOTCH4 does not appear to have a known, major role in bone formation; however other NOTCH genes are involved in the proliferation and maturation of chondrocytes [35].

Extending our investigation of the biological differences between these two dura classes, we examined genes outside of these pathways and identified several genes that play key roles in biological processes related to endochondral ossification, which is the process by which bones in the cranial base are formed. Although these genes were not within our top 100 ranked genes identified from the clustering analysis, they are still among some of the most significantly differentially expressed genes between the two classes. Examples of these include the runt-related transcription factor 2 (RUNX2), runt-related transcription factor 3 (RUNX3), collagen, type II, alpha 1 (COL2A1), parathyroid hormone 1 receptor (PTH1R), and notch 1 (NOTCH1) [35]. With the exception of COL2A1, all of these genes were down-regulated in class 2 compared to class 1. Another strong biological candidate is transforming growth factor, beta receptor II (TGFBR2) which was expressed at significantly lower levels in class 2 patients. A previous study generated mice with a conditional deletion of TGFBR2 in COL2A1 expressing cells and found defects in the cranial base and vertebrae [36]. Two additional studies in mice demonstrated that the conditional inactivation of TGFBR2 specifically in mesoderm-derived cells results in defects in the supraoccipital bone and C1 vertebra and meningoencephalocele [37], whereas TGFBR2 inactivation in neural crest cells leads to calvaria defects and cleft palate [38]. This report has particular relevance as a shortened supraoccipital bone and reduced TGFBR2 expression was observed in class 2 patients. Taken together, these findings implicate several strong biological candidates pertinent to endochondral ossification and suggest that the dura mater may be a reasonable tissue to examine for CMI.

Although encouraged by our findings, there are several important limitations to present. First, our relatively small sample size resulted in limited power for the clustering analysis and follow-up characterization. Obtaining clinical tissue samples from CMI patients is extremely challenging as they are not readily available thus greatly inhibiting the collection of a large sample size. Despite our limited power, we did identify significant underlying structure within the datasets, some of which appeared to be biologically relevant. However, many of the analyses would not have withstood a correction for multiple testing. As one of the primary purposes of this study was to generate hypotheses, we feel it appropriate to examine our results without such an adjustment noting that future work and continued ascertainment would be needed to validate any findings. Another limitation of our study relates to the acquisition of disease relevant patient tissue for gene expression analysis. Our study is not unique in its challenges to ascertain appropriate biological samples for a developmental disease, particularly with respect to developmental stage. Thus, we cannot disregard the implications of this when interpreting our findings. However, we did identify several strong biological candidates from the dura analysis providing additional support for its potential relevance. Additionally, due to the small size of the dura samples acquired and the fact that dura is largely comprised of collagen bundles, low RNA yield was obtained resulting in a limited number of samples with RNA remaining for validation experiments. Moreover, due to the unique nature of this study, we are currently unable to replicate our findings using an independent study population. While further validation and replication of our results is vital, our goal by reporting these initial findings is to encourage additional research in this understudied field and the generation of similar datasets to be used for replication and further investigation into the underlying disease biology. Finally, as the underlying biologic mechanism for CMI is currently unknown, it is unclear which tissue at this stage in development would be most biologically relevant. Thus, future studies using other tissue types, including bone and/or the pia-arachnoid layer may further elucidate the biology of CMI and perhaps correlate better with CMI symptoms or structure.

## Conclusions

This study is unique as it represents the first study of its kind for CMI where multiple biological and radiological datasets exist across the same set of patients thereby motivating the extension of the original sparse k-means clustering method to accommodate multiple datasets from different sources. By applying both clustering methods to our data, we were able to establish patient classes based solely on biological or radiological data, as well as through the integration of these datasets. Although further validation and replication is needed, examination of the genes differentially expressed between the dura classes implicated several strong biological candidates for future investigation. Biological characterization of the blood classes identified a gene expression profile that corresponded to a global down-regulation in protein synthesis.

## Abbreviations

CMI: Chiari Type I Malformation; PF: Posterior fossa; OSA: Ordered subset analysis; RIN: RNA Integrity number; RT-qPCR: Real-time quantitative PCR; CV: Coefficient of variation; PCA: Principal components analysis; BCSS: Between cluster sum of squares; CI: Confidence interval; Dura-Cranial: Dura gene expression and PF trait data; Blood-Cranial: Blood gene expression and PF trait data; Blood-Dura-Cranial: Blood gene expression, dura gene expression, and PF trait data; rDNA: Ribosomal DNA; ASD: Autism spectrum disorder.

## Competing interests

A potential conflict of interest exists. Dr. Allison Ashley-Koch is chair of the Chiari and Syringomyelia Foundation (CSF) scientific, education, and advisory board. CSF provided partial funding for this study, as well as salary support for Dr. Christina Markunas. The funders had no role in study design, data collection and analysis, decision to publish, or preparation of the manuscript.

## Authors’ contributions

CAM participated in the conception and design of the study, performed some of the molecular work (RNA extractions and ran Illumina HT-12 chips), collected some of the patient samples, performed the statistical analyses, interpreted the results, and drafted the paper. EL participated in the analytical design of the study and conceived of, developed code, and drafted the methods section describing the integrative sparse k-means clustering algorithm. KS performed some of the molecular work (Ran Illumina HT-12 chips and RT-qPCR experiments) and collected some of the patient samples. HC ascertained the patients and collected some of the patient samples. CCD performed some of the molecular work (Participated in some of the RT-qPCR experiments). DSE reviewed and confirmed all MRI measurements. GG performed some of the surgeries and helped ascertain patients. HF performed some of the surgeries and helped ascertain patients. AAK participated in the conception and design of the study, collected some of the patient samples, interpreted the results, co-supervised the study, and drafted the manuscript. SGG participated in the conception and design of the study, collected some of the patient samples, interpreted the results, co-supervised the study, and drafted the manuscript. All authors read, provided critical feedback, and approved the final manuscript.

## Pre-publication history

The pre-publication history for this paper can be accessed here:

http://www.biomedcentral.com/1755-8794/7/39/prepub

## Supplementary Material

Additional file 1 Detailed description of the integrative sparse k-means clustering approach. Click here for file

Additional file 2 Class assignments for all samples from each sparse k-means clustering analysis. Click here for file

Additional file 3 Biological, radiological, and clinical characterization of the remaining classes. Click here for file

Additional file 4 Real-time quantitative PCR results. Click here for file
