# Supplementary material for: Identification of Chiari Type I Malformation subtypes using whole genome expression profiles and cranial base morphometrics
Source: BMC Med Genomics. 2014 Jun 25;7:39. doi: 10.1186/1755-8794-7-39 (PMC4082616; doi:10.1186/1755-8794-7-39)
Supplement: Additional file 1 — Detailed description of the integrative sparse k-means clustering approach. [file 1755-8794-7-39-S1.pdf]

Integrative Sparse K-means Clustering

Here we give a detailed description of the integrative sparse k-means approach to clustering multiple data sources. The approach is a straightforward extension of the sparse k-means method to cluster a single data source that is described in [1], and we borrow much of the notation therein.

Let $X : p \times n$ be a single data source with p features and n objects to be clustered. Let Θ be a partition of the objects into K clusters, and let $\bar{x}_{jk}$ be the arithmetic mean of the objects belonging to cluster k ($k \in \{1, \ldots, K\}$) for feature j ($j \in \{1, \ldots, p\}$). The standard sparse k-means clustering algorithm then determines Θ by maximizing the weighted between cluster sum of squares (BCSS)

$$\text{BCSS} = \sum_{j=1}^{p} w_j \sum_{k<k'} (\bar{x}_{jk} - \bar{x}_{jk'})^2$$

subject to $||\mathbf{w}||^2 \leq 1$, $w_j \geq 0$ for each j, and $||\mathbf{w}||_1 = \sum_{j=1}^{p} w_j \leq s$. A large weight w_j indicates that the j'th feature contributes strongly to the clustering. If the value of the tuning parameter s is small some of the w_j will shrink to 0 and those features will not be involved in the clustering.

We extend the above framework to accommodate m data sources $X_1 : p_1 \times n, \ldots, X_m : p_m \times n$, where n is the number of objects to be clustered (common to all data sources) and p_i is the number of features in data source i. Let $\bar{x}_{ijk}$ be the arithmetic mean of the objects belonging to cluster k ($k \in \{1, \ldots, K\}$), for feature j ($j \in \{1, \ldots, p_i\}$) of data source i ($i \in \{1, \ldots, m\}$). The integrative sparse k-means clustering algorithm then determines a partition Θ by maximizing the weighted between cluster sum of squares

$$\text{BCSS} = \sum_{i=1}^{m} \sum_{j=1}^{p_i} w_{ij} \sum_{k<k'} (\bar{x}_{ijk} - \bar{x}_{ijk'})^2$$

subject to

- $||\mathbf{w_i}||^2 \leq 1$ for $i = 1, \ldots, m$.
- $w_{ij} \geq 0$ for all i, j.
- $||\mathbf{w_i}||_1 = \sum_{j=1}^{p_i} w_{ij} \leq s_i$

Note that this framework allows for a different tuning parameter for each data source (as given by the s_i's). Choosing $s_i = 1$ corresponds to perfect sparsity

for data source i (just one feature selected) and choosing $s_i = \sqrt{p_i}$ corresponds to no sparsity (all features included). To reduce the complexity of choosing m different sparsity parameters we can instead choose a single α, $0 < \alpha < 1$ and define $s_i = \alpha\sqrt{p_i}$ for each i. Here α adjustes the sparsity level for all data sources, relative to the number of features in each source.

The integrative clustering framework is potentially unbalanced, as the number of features or the amount of variablity in a data source can potentially affect the degree to which it influences the clustering. Hence, we use a default procedure to normalize each data source before clustering. This normalization procedure follows three steps:

1. Center by subtracting the mean within each feature (this will not affect k-means clustering, but simplifies the normalization process).

2. Scale each feature by dividing by its standard deviation.

3. Divide all of the values in data source i by $p_i^{1/4}$.

This procedure ensures that the total weighted sum of squares in each data source,

$$\sum_{j=1}^{p_i} w_{ij} x_{ij}^2$$

are equal for any $\mathbf{w_1}, \ldots, \mathbf{w_m}$ that satisfy the constraints above. Note that the total sum of squares is the between cluster sum of squares plus the within cluster sum of squares.

To choose the number of clusters K and the tuning parameters s_i (or α) for integrative clustering we use the gap statistic in a way that is analogous to the approach described in [1]. However, to compute the gap statistic we permute the objects within each data source, rather than within each feature. This is because we would like to identify clusters that are significantly expressed on multiple data sources, rather than multiple features in a single data source.

References

[1] Witten, DM and Tibshirani, R. A framework for feature selection in clustering. *Journal of the American Statistical Association* **105(490)**: 713-726.
